# Systemic Lupus Erythematosus With Acute Inflammatory Demyelinating Polyneuropathy in Pregnancy: A Rare Multisystem Presentation

**DOI:** 10.7759/cureus.97586

**Published:** 2025-11-23

**Authors:** Hadia Saeed, Mustafeez Ur Rehman

**Affiliations:** 1 Emergency Department, King's College Hospital NHS Foundation Trust, London, GBR; 2 Internal Medicine Department, Manchester University NHS Foundation Trust, Manchester, GBR

**Keywords:** acute inflammatory demyelinating polyradiculoneuropathy, lupus myocarditis, lupus nephritis, neurofascin antibodies, pregnancy-related complications, systemic lupus erythematosus

## Abstract

Systemic lupus erythematosus (SLE) is a chronic autoimmune disease associated with significant maternal and fetal risks during pregnancy. Lupus nephritis further increases morbidity, while neurological complications are less common. Acute inflammatory demyelinating polyneuropathy (AIDP), a variant of Guillain-Barré syndrome, is exceptionally rare in pregnancy. We describe a 24-year-old primigravida at 14 weeks of gestation who presented with progressive lower limb weakness, numbness, and diplopia. Laboratory tests demonstrated proteinuria and hypoalbuminemia, which were initially attributed to pregnancy. Forty-eight hours later, she re-presented with worsening weakness and inability to walk. Neurological assessment, cerebrospinal fluid analysis, nerve conduction studies, and strongly positive pan-neurofascin antibodies confirmed AIDP. Further evaluation revealed nephrotic syndrome secondary to class III lupus nephritis. During immunosuppressive therapy, including tacrolimus, she developed lupus-related myocarditis with reduced ejection fraction. The pregnancy was further complicated by intrauterine growth restriction, oligohydramnios, and preeclampsia, necessitating emergency cesarean section at 27 + 1 weeks. Both mother and infant survived with ongoing multidisciplinary follow-up. This case highlights an exceptionally rare multisystem presentation of SLE in pregnancy, involving lupus nephritis, AIDP, and myocarditis. Early recognition and coordinated multidisciplinary management are essential to optimize maternal and fetal outcomes in such complex cases.

## Introduction

Systemic lupus erythematosus (SLE) is an autoimmune disorder characterized by chronic inflammation affecting multiple organ systems. It predominantly affects women of childbearing age, and pregnancy in patients with SLE is associated with increased maternal and fetal morbidity and mortality [1,2]. Lupus nephritis is a serious complication of SLE, often resulting in proteinuria, hypoalbuminemia, and impaired renal function. Active lupus nephritis during pregnancy increases the risk of preeclampsia, preterm delivery, and fetal growth restriction [3,4].

Neurological involvement in SLE is common, presenting most frequently as seizures, cognitive impairment, cerebrovascular accidents, or peripheral neuropathies. Acute inflammatory demyelinating polyneuropathy (AIDP), a variant of Guillain-Barré syndrome, is an uncommon neurological manifestation in SLE, and reports of its occurrence in pregnancy are extremely rare [5,6]. Recent studies suggest that pan-neurofascin antibodies are associated with severe autoimmune neuropathies, although their presence in pregnant patients has been seldom described [7].

Immunosuppressive therapies, including corticosteroids, rituximab, and calcineurin inhibitors such as tacrolimus, are commonly used to manage lupus nephritis, even during pregnancy. While effective, these treatments carry potential adverse effects. Lupus-induced myocarditis is exceptionally rare but clinically significant [8].

## Case presentation

A 24-year-old primigravida at 14 weeks of gestation presented to the emergency department with a five-day history of progressive bilateral hand and foot numbness, lower limb weakness, difficulty climbing stairs, frontal headache, hemoptysis, and left-sided diplopia. She had recently returned from a Caribbean cruise visiting several locations, including the American and British Virgin Islands and the Dominican Republic. She reported no fever, diarrhea, or recent infections. There was no personal or family history of neurological or autoimmune disease.

On initial assessment, she was alert, oriented, and hemodynamically stable. Neurological examination revealed no focal deficits. Blood tests demonstrated hypoalbuminemia and proteinuria. Given her early gestation, these findings were initially attributed to pregnancy-related changes. She was discharged with advice for midwife follow-up and outpatient monitoring.

Forty-eight hours later, she re-presented with worsening weakness, bilateral leg swelling, and inability to stand independently. Neurological examination demonstrated absent deep tendon reflexes in all four limbs and reduced muscle strength (Medical Research Council grade 3/5 in the lower limbs and 4/5 in the upper limbs). Mild sensory deficit was noted below the knees and elbows, more prominent on the right side. Cranial nerve examination was normal. CT head was unremarkable.

Neurology consultation suspected Guillain-Barré syndrome and Lyme disease. Lumbar puncture revealed albuminocytologic dissociation, with elevated protein and normal cell count. Nerve conduction studies demonstrated demyelinating neuropathy consistent with AIDP. Serum pan-neurofascin antibodies were strongly positive (NF155 1:1600, NF186 1:2400, IgG1), supporting an autoimmune neuropathy diagnosis. She received a standard course of intravenous immunoglobulin over five days, with no significant improvement in limb strength. Comprehensive infectious disease screening, including testing for Lyme disease, yielded negative results.

**Figure 1:**
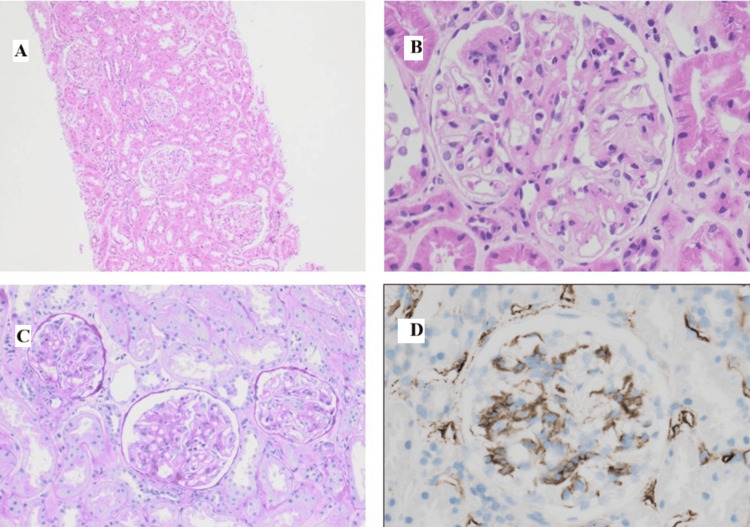
Histopathological images of renal biopsy showing features consistent with lupus nephritis (ISN/RPS Class III) (A) H&E stain at low magnification (×200) showing glomeruli with mild mesangial hypercellularity. (B) H&E stain at high magnification (×400) demonstrating segmental endocapillary hypercellularity and karyorrhexis. (C) PAS stain (×400) highlighting mesangial matrix expansion and focal basement membrane thickening. (D) Immunohistochemistry for C1q (×400) showing granular mesangial and capillary wall deposits H&E: hematoxylin and eosin; PAS: Periodic acid-Schiff

Further evaluation identified nephrotic-range proteinuria (urine protein-creatinine ratio >3 g/day) and hypoalbuminemia. Renal biopsy confirmed WHO class III lupus nephritis (Figure 1). Autoimmune serology revealed antinuclear antibody 1:1,280 (homogeneous), double-stranded DNA positive, Ro52/60 positive, La positive, Sm and RNP positive, and low complement levels (C3 and C4) consistent with SLE. A multidisciplinary review recommended immunosuppressive therapy, including rituximab, tacrolimus, azathioprine, hydroxychloroquine, and prednisolone. Obstetric management included low-dose aspirin and nifedipine for preeclampsia prophylaxis and blood pressure control.

**Figure 2:**
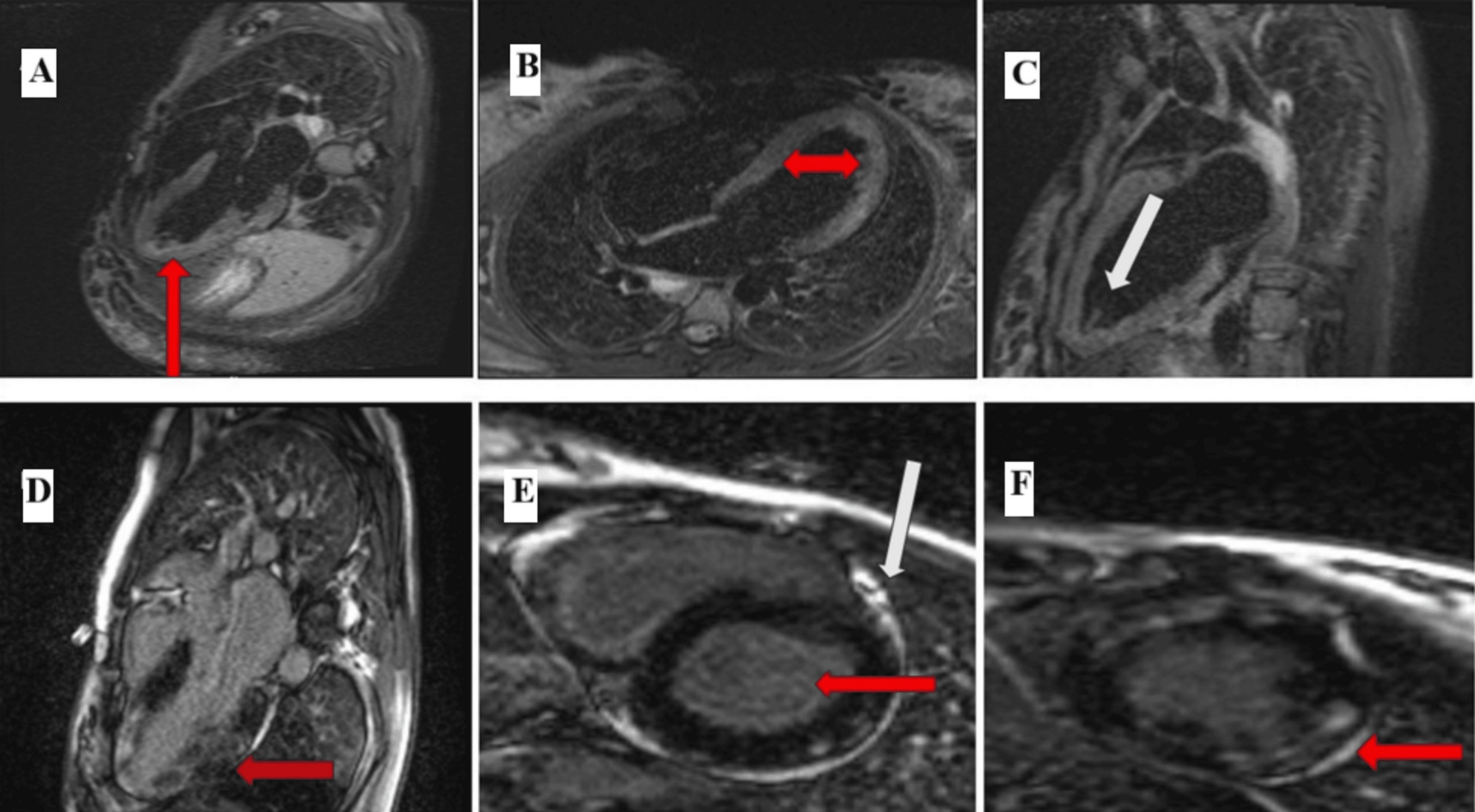
Cardiac magnetic resonance imaging (A-C) T2-weighted images; long axis with three-chamber, four-chamber, and two-chamber views showing evidence of significant active inflammation/edema involving predominantly the lateral wall and all mid distal segments, with the highest values toward the apex. (D) Long-axis view T2-weighted image showing left ventricular inflow-outflow plane. Arrow indicates a patchy intramyocardial gadolinium uptake pattern in the lateral wall and all mid-distal segments with fibrosis consistent with significant active myocarditis. (E,F) Short-axis view with late gadolinium enhancement from subepicardial to mid-wall in the basal distal lateral, mid-distal septal, and other apical segments, becoming near transmural in the distal segments

During her inpatient stay, the patient developed tachycardia. ECG demonstrated T-wave inversions. Serial troponin I levels peaked at 11,084 ng/L. Transthoracic echocardiography revealed a reduced left ventricular ejection fraction (LVEF; 46%) with apical hypokinesia. Cardiac MRI demonstrated myocardial edema, mild pericardial effusion, and preserved LVEF of 54%, consistent with myocarditis (Figure 2), suspected secondary to lupus and later confirmed on myocardial biopsy. Bisoprolol therapy was initiated.

Serial obstetric ultrasounds demonstrated significant intrauterine growth restriction (less than the third percentile), oligohydramnios, and elevated uterine artery pulsatility index. At 27 + 1 weeks of gestation, the patient underwent emergency cesarean section due to severe fetal compromise and maternal preeclampsia. The neonate required NICU admission for prematurity and growth restriction but stabilized. The mother’s neurological and cardiac status gradually improved. She was discharged after three days with ongoing multidisciplinary follow-up and immunosuppressive medications, including azathioprine, hydroxychloroquine, prednisolone, and tacrolimus.

## Discussion

This case highlights a unique and complex presentation of SLE in pregnancy, with concurrent lupus nephritis (Figure 1), AIDP with pan-neurofascin antibodies, and lupus-induced myocarditis (Figure 2). It illustrates the diagnostic challenges and therapeutic considerations required for optimal maternal and fetal outcomes. The aim of the study is to indicate diagnostic problems in pregnant patients with new onset SLE in whom neurological symptoms are present at the time of diagnosis of SLE, but also with complications that occurred after diagnosis.

Lupus nephritis (Figure 1) during pregnancy increases maternal and fetal morbidity, with higher rates of preeclampsia, preterm birth, and fetal growth restriction [1,3,6]. Neurological involvement in SLE is common but usually presents as seizures, cerebrovascular events, or cognitive impairment. AIDP is rare, and its occurrence in pregnancy is exceedingly uncommon [2,8]. The presence of pan-neurofascin antibodies in this patient indicates a severe autoimmune neuropathy [4].

Immunosuppressive therapy is essential to manage lupus nephritis (Figure 1) and AIDP but carries potential adverse effects. Multidisciplinary collaboration among rheumatology, neurology, cardiology, and obstetrics teams was central to achieving favorable maternal and fetal outcomes. This case underscores the importance of early recognition, timely investigation, and coordinated management of complex multisystem complications in pregnant patients with SLE.

## Conclusions

SLE can present with rare multisystem complications during pregnancy, including lupus nephritis, AIDP, and myocarditis. Early recognition, timely investigation, and multidisciplinary care are critical to optimizing maternal and fetal outcomes. Vigilant monitoring for neurological, renal, cardiac, and obstetric complications is essential in pregnant patients with SLE receiving immunosuppressive therapy.

Pregnancy can mask or mimic systemic autoimmune conditions such as SLE/AIDP, leading to diagnostic challenges. Peripheral neuropathy in pregnancy should prompt consideration of autoimmune and inflammatory causes, not just nutritional or gestational factors.

## References

[1] Lateef A, Petri M (2013). Managing lupus patients during pregnancy. Best Pract Res Clin Rheumatol.

[2] Pathmanathan U, Sivakumaran S, Eccles-Smith J, Craven AM, Smith JP, Puri P, Hepburn K (2025). New diagnosis of lupus nephritis in the third trimester-a case report. Nephrology (Carlton).

[3] Gasparotto M, Gatto M, Binda V, Doria A, Moroni G (2020). Lupus nephritis: clinical presentations and outcomes in the 21st century. Rheumatology (Oxford).

[4] Zeeman GG (2001). A case of acute inflammatory demyelinating polyradiculoneuropathy in early pregnancy. Am J Perinatol.

[5] Hughes RA, Cornblath DR (2005). Guillain-Barré syndrome. Lancet.

[6] Zafar MS, Naqash MM, Bhat TA, Malik GM (2013). Guillain-Barré syndrome in pregnancy: an unusual case. J Family Med Prim Care.

[7] Doppler K, Stengel H, Appeltshauser L, Grosskreutz J, Man Ng JK, Meinl E, Sommer C (2018). Neurofascin-155 IgM autoantibodies in patients with inflammatory neuropathies. J Neurol Neurosurg Psychiatry.

[8] Ergin RN (2014). Pregnancy-associated systemic lupus erythematosus. Proc (Bayl Univ Med Cent).

